# A Unique Urinary Metabolic Feature for the Determination of Bladder Cancer, Prostate Cancer, and Renal Cell Carcinoma

**DOI:** 10.3390/metabo11090591

**Published:** 2021-09-02

**Authors:** Sujin Lee, Ja Yoon Ku, Byeong Jin Kang, Kyung Hwan Kim, Hong Koo Ha, Suhkmann Kim

**Affiliations:** 1 Department of Chemistry and Chemistry Institute for Functional Materials, Institute for Plastic Information and Energy Materials, Pusan National University, Busandaehak-ro 63, Geumjeong-gu, Busan 46241, Korea; isujin@pusan.ac.kr; 2 Department of Urology, Dongnam Institute of Radiological & Medical Sciences Cancer Center, Busan 46033, Korea; pnumed@pusan.ac.kr; 3 Department of Urology, College of Medicine, Pusan National University, Busan 49241, Korea; uropean86@gmail.com (B.J.K.); bravekim80@naver.com (K.H.K.); 4 Department of Urology, College of Medicine, Pusan National University and Biomedical Research Institute, Pusan National University Hospital, Busan 49241, Korea; hongkooha@pusan.ac.kr

**Keywords:** urological cancer, metabolomics, NMR spectroscopy, urine, discriminant metabolites

## Abstract

Prostate cancer (PCa), bladder cancer (BCa), and renal cell carcinoma (RCC) are the most prevalent cancer among urological cancers. However, there are no cancer-specific symptoms that can differentiate them as well as early clinical signs of urological malignancy. Furthermore, many metabolic studies have been conducted to discover their biomarkers, but the metabolic profiling study to discriminate between these cancers have not yet been described. Therefore, in this study, we aimed to investigate the urinary metabolic differences in male patients with PCa (*n* = 24), BCa (*n* = 29), and RCC (*n* = 12) to find the prominent combination of metabolites between cancers. Based on ^1^H NMR analysis, orthogonal partial least-squares discriminant analysis was applied to find distinct metabolites among cancers. Moreover, the ranked analysis of covariance by adjusting a potential confounding as age revealed that 4-hydroxybenzoate, *N*-methylhydantoin, creatinine, glutamine, and acetate had significantly different metabolite levels among groups. The receiver operating characteristic analysis created by prominent five metabolites showed the great discriminatory accuracy with area under the curve (AUC) > 0.7 for BCa vs. RCC, PCa vs. BCa, and RCC vs. PCa. This preliminary study compares the metabolic profiles of BCa, PCa, and RCC, and reinforces the exploratory role of metabolomics in the investigation of human urine.

## 1. Introduction

Urological cancers include tumors of the kidney, bladder, and in men, also of the prostate and testis. Among these, prostate cancer (PCa), bladder cancer (BCa), and renal cell carcinoma (RCC) are the most frequent cancer types. In the United States of America, an estimated 191,930 new cases and 33,330 deaths from PCa and 62,110 new cases and 13,050 deaths from BCa have been reported [1]. In most cases, the early symptoms of urologic cancer do not appear until the cancer has progressed, and because the incidence is higher in men than women, these cancers are considered to be dangerous cancers for men. In addition, there are no cancer-specific clinical symptoms between PCa, BCa, and RCC. Urological cancers are accompanied by common symptoms such as dysuria, hematuria, and frequent urination, which may be a sign of cancer [2]. However, these symptoms can also appear with urinary tract infection [3]. Thus, diagnostic methods such as cystoscopy of the bladder, digital rectal examination of the prostate including biopsies, and computed tomography (CT) scans are needed to distinguish the three representative urological malignancies. However, these diagnoses are very invasive and require radiation exposure, rendering them unpleasant to patients as well as risking unnecessary complication [4,5,6]. Therefore, although these examinations remain the optimal norm for detecting urological cancers, given their invasiveness and high cost, non-invasive approaches for early discrimination between three urological cancers are essential.

Metabolomics involves the comprehensive systemic profiling of metabolite concentrations and their cellular perturbations that can be influenced by numerous factors, such as genetics, pathological conditions, and external factors (e.g., diet, lifestyle, and environment) [7]. This technique is closely related to the phenotype in biological systems, so is increasingly being applied toward a potential diagnostic tool to monitor disease status [8,9,10]. The main analytical platforms for metabolic profiling are nuclear magnetic resonance (NMR) spectroscopy and mass spectrometry (MS). Both approaches are appropriate for metabolic analysis, but they have different analytical strengths and weaknesses [7]. MS can identify metabolites with high sensitivity. However, the matrix effect and ionization suppression can be influenced by the presence of other chemical compounds, resulting in discrepancies [11]. In addition, this requires extensive sample preparation and consequently results in sample disintegration. By contrast, although NMR has low sensitivity, it provides high reproducibility. The sample preparation is straightforward and non-destructive, enabling further re-analysis of the same sample [11,12,13]. Using these approaches, cancer-related alterations of metabolic pathway as well as molecular markers could be discovered. Bansal et al. explored the metabolic differences between the serum of BCa patients and that of healthy controls using NMR, and they proposed that the combination of dimethylamine, malonate, lactate, glutamine, histidine, and valine could differentiate BCa from the control [14]. Ming Cao et al. showed that the serum metabolic profile of BCa patients was distinctly different from that of the calculi and healthy individuals using NMR spectroscopy. They reported a decrease in the levels of isoleucine/leucine, tyrosine, lactate, glycine, and citrate as well as an increase in those of lipids and glucose. They offered potentially valuable information for the pathogenic process of BCa and a noninvasive method for the diagnosis of the disease [15]. In addition, sarcosine (*N*-methylglycine) levels, as a well-known biomarker of prostate cancer, was elevated during prostate cancer progression compared to very low levels in a healthy control [16]. In an NMR-based metabolomics study, several metabolites including citrate, myo-inositol, and spermine was identified as tentative biomarkers of prostate cancer [17,18]. Furthermore, in metabolomics study for renal cell carcinoma, Monteiro et al. defined 32 metabolite markers that clearly discriminate RCC patients from healthy controls by principal component analysis [19].

From these many previous studies, it has been proven that NMR-based metabolomics is a superior methodology for clinical diagnosis of various disease. On the other hand, these metabolic studies in urological oncology have been focused on biomarker discovery by comparing the control group of healthy subjects, whereas the studies about metabolic comparison among bladder, prostate cancer, and renal cell carcinoma have not yet been described. In this study, using NMR spectroscopy, we mainly aim to compare the metabolic differences between BCa, PCa, and RCC and to find the discriminant metabolites among cancer groups. Therefore, the purpose of our study is not to determine whether or not cancer is cancerous compared to a healthy control, but to differentiate between urological cancer groups in metabolomics. Furthermore, through this preliminary study, we expect to provide a faster determination of urological cancer types in patients with voiding dysfunction.

## 2. Results and Discussion

### 2.1. Study Population Characteristics

In the present study, a total of 65 patients were classified into the following groups: 29 patients with BCa, 24 patients with PCa, and 12 patients with RCC. The mean age of the patients with BCa was 67.92 ± 10.42 years (range, 58–79 years), and those of the patients with PCa and RCC were 68.21 ± 5.54 years (range, 59–79 years) and 62.17 ± 11.76 years (range, 34–78 years), respectively. All subjects in BCa, PCa, and RCC were matched for sex. On the other hand, serum creatinine (SCr) and glomerular filtration rate (eGFR) of BCa, PCa, and RCC were observed to be less than 1.2 mg/dL and more than 60 mL/min/1.73 m^2^, respectively, which were normal. These results mean that the renal function of the patients in each group is normal [20]. In the BCa group, 15/29 (48.27%) were classified as low grade by WHO/ISUP classification. The majority of the patients with BCa were found to be stage Ta (44.83%) and T1 (37.93%) at the final pathology, followed by T2 (15.15%) and Tis (6.89%). Among the 24 patients with PCa, 5 (20.9%), 3 (12.5%), 8 (33.3%), and 8 (33.3%) had clinical Gleason scores of 6, 7, 8, and above 8, respectively, upon preoperative biopsy. A total of 8 and 16 urine samples were collected for hormone-sensitive prostate cancer (HSPC) and castration-resistant prostate cancer (CRPC), respectively. The patients with PCa were found to be stage T1 (12.5%), T2 (25.0%), T3 (58.33%), and T4 (4.17%) at the final pathology. In the RCC group, 8/12 (66.67%) patients were classified as low-grade (Fuhrman grade 2). Furthermore, 7/12 (58.33%) patients were pT1 stage, followed by T3 (4/12, 33.33%), and T4 (1/12, 8.33%) stages. The detailed clinical characteristics of the patients are presented in Table 1.

### 2.2. Metabolic Differences for Urological Cancers by Using Multivariate Pattern Recognition Analysis

**Figure 1:**
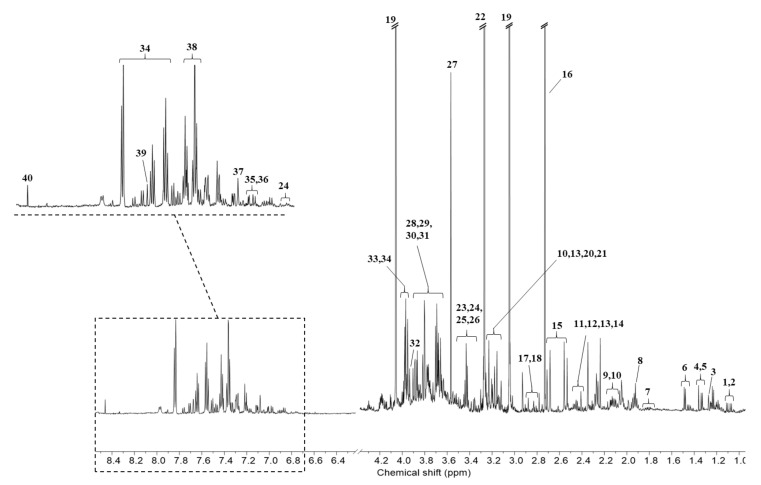
Representative ^1^H NMR Spectrum with water suppression of urological cancer. (1) Valine, (2) 3-Hydroxyisobutyrate, (3) 3-Hydroxyisovalerate, (4) 2-Hydroxyisobutyrate, (5) Lactate, (6) Alanine, (7) Lysine, (8) Acetate, (9) Methionine, (10) O-Acetylcarnitine, (11) Pyroglutamate, (12) Succinate, (13) Carnitine, (14) Glutamine, (15) Citrate, (16) Dimethylamine, (17) *N,N*-Dimethylglycine, (18) *N*-Methylhydantoin, (19) Creatinine, (20) cis-Aconitate, (21) Choline, (22) Trimethylamine N-oxide, (23) Taurine, (24) 4-Hydroxyphenylacetate, (25) Glucose, (26) myo-Inositol, (27) Glycine, (28) Arabinitol, (29) Gluconate, (30) Mannitol, (31) Betaine, (32) Creatine, (33) Glycolate, (34) Hippurate, (35) Tyrosine, (36) 4-Hydroxybenzoate, (37) Histidine, (38) N-Phenylacetylglycine, (39) Pseudouridine, (40) Formate. The region of water and urea was excluded.

Representative ^1^H NMR spectra with water suppression obtained from the urine of patients with PCa, BCa, and RCC are shown in Figure 1.

A total of 40 metabolites were commonly identified in urinary cancer groups and quantified using Chenomx suite 8.4 NMR 600 MHz library based on human metabolome database (HMDB) database. In addition, to accurately assign the resonances of metabolites, literature data and public databases, such as Pubchem were used [21]. To explore the possible differences in the metabolic profiles, a multivariate statistical analysis for the three groups was performed by applying supervised method, partial least squares discriminant analysis (PLS-DA) and orthogonal partial least squares discriminant analysis (OPLS-DA) score plot, which require prior knowledge of sample clustering for the purpose of discriminant analysis and are used to elucidate the most reliable variables for group separation [22,23]. 

**Figure 2:**
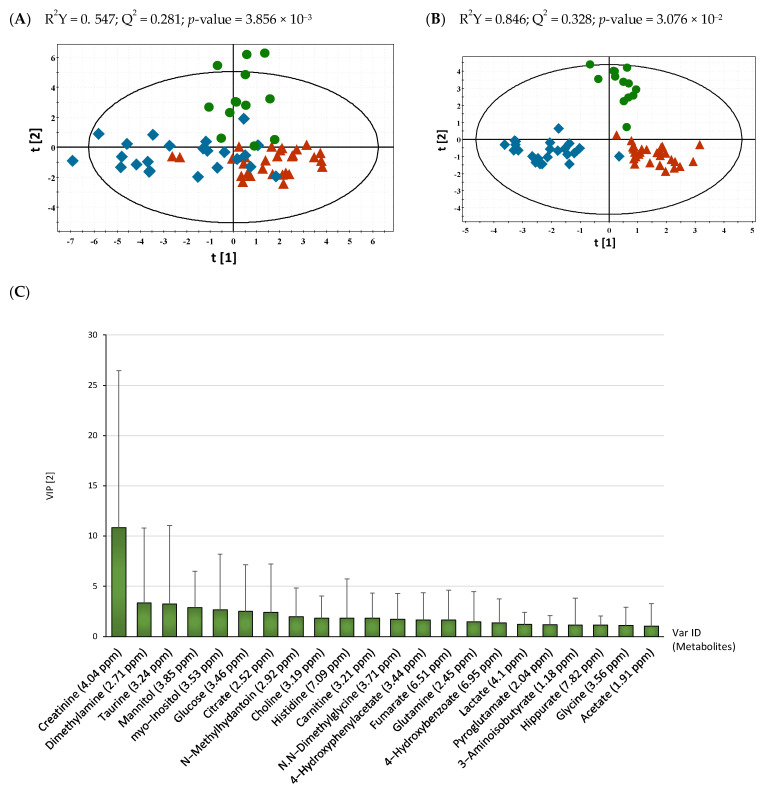
Metabolic multivariate pattern recognition analysis. (**A**) PLS-DA score plots from the ^1^H NMR spectra with water suppression from patients with urological cancers: BCa (*n* = 29, ▲ red); PCa (*n* = 24, ◆, blue); RCC (*n* = 12, ●, green); (**B**) OPLS-DA score plots based ^1^H NMR spectra of urinary metabolites among cancer groups. All the score plots were assessed by R^2^Y, Q^2^, and *p*CV-ANOVA (*p* < 0.05); (**C**) VIP score plot represents the variables with VIP value higher than 1 selected in the OPLS-DA model. Var ID indicates each contributing metabolite.

The PLS-DA model which was built using the two principal components showed the overall discriminatory pattern among BCa, PCa, and RCC. This model was assessed by the goodness of fit R^2^Y = 0.547 and predictive quality Q^2^ = 0.281 (Figure 2A). For improved interpretability, we preferred the OPLS-DA model which filters out the structured noise in the data set, uncorrelated to the major discriminating response, thus reducing the complexity of the model [23]. As shown in Figure 2B, we observed a clear separation among the three urological groups compared with the PLS-DA model. This OPLS-DA showed a proper fitting of data (R^2^Y value = 0.846) and predictability (Q^2^ value = 0.328). The significance of these classification models was assessed by cross-validated predictive residuals *p*(CV-ANOVA) (estimated by *p*-value < 0.05). Furthermore, the differences of metabolic pattern between intergroups were identified. The OPLS-DA models of BCa vs. RCC (Figure S1A) and PCa vs. RCC (Figure S1B) showed good predictability, whereas the model of PCa vs. BCa showed lower predictability compared to other classification models, which indicated a low metabolic difference between PCa and BCa (Figure S1C).

To select significant metabolites that mostly contributed to the differences among the three groups, the variable selection method (VIP) was applied to the OPLS-DA model based on t [2] axis. Generally, a cutoff for VIP higher than 1 is regarded as proper [24], therefore 22 metabolites with VIP > 1 were selected as influential metabolites in separation of groups (Figure 2C). Particularly, creatinine showed the highest value, followed by dimethylamine, taurine, mannitol, myo-inositol, glucose, citrate, and *N*-methylhydantoin. Studies based on NMR spectroscopy for the detection of metabolite markers of prostate cancer have mainly been performed on prostate biopsy tissue or prostatic secretion. From these studies, myo-inositol, citrate, and choline-containing compounds were reported as potential markers of PCa [18,25,26]. In addition, other urinary metabolomics studies of BCa suggested that carnitine, pyroglutamate, pseudouridine, histidine, and hippurate could be putative biomarkers [27,28,29,30,31]. Similarly, in RCC, Monteiro et al. revealed 32 urinary signature metabolites containing 4-hydroxyphenylacetate and hippurate to be excreted at lower levels in RCC [19]. Furthermore, Kim et al. showed that quinolinate, 4-hydroxybenzoate, and genistate were significantly differentially expressed in RCC patients compared with the control group [32]. Taking into consideration the accumulating evidence regarding the urinary metabolomics of urological cancers, we hypothesized that the 22 metabolites with VIP values higher than 1 identified in this study could be considered as tentative discriminatory variables of BCa, PCa, and RCC.

### 2.3. Comparison of Differential Metabolites Levels among Urological Cancers Using Univariate Statistical Analysis

Quantified metabolites were normalized by total area to minimize the dilution effect of urine among groups. The median value and interquartile range (IQR) of the normalized metabolite concentration for BCa, PCa, and RCC are presented in Table S1.

**Figure 3:**
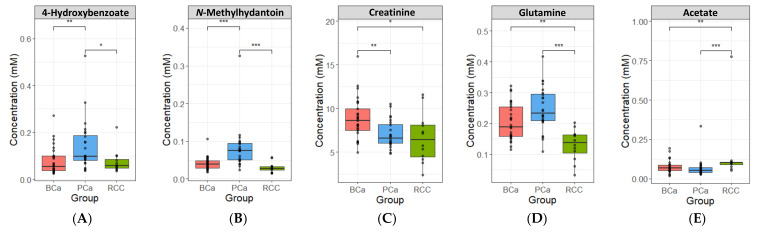
Boxplots of metabolites concentrations that were significantly different among BCa, PCa, and RCC from non-parametric analysis of covariance (Ranked ANCOVA). (**A**) 4-hydroxybenzoate (4-HBA), (**B**) *N*-methylhydantoin (*N*-MH), (**C**) creatinine, (**D**) glutamine, and (**E**) acetate. Each dot in the boxes represents an individual sample concentration. * <0.05, ** <0.01, *** <0.001.

Before identifying the metabolites, which distinguish the three urological cancers, the normal distribution of the urinary metabolic profile dataset was examined using the Kolmogorov-Smirnov test, which showed that the samples under analysis did not exhibit normal distribution (*p* < 0.05) within each group (Table S1). Then, an age-adjusted non-parametric analysis of covariance (Ranked ANCOVA) was used to determine significant metabolites among the groups with a Bonferroni correction method (adjusted *p*-value < 0.05) (Table S1) [33]. A total of five metabolites were differentially expressed in urological cancer groups: 4-hydroxybenzoate (4-HBA), *N*-methylhydantoin (*N*-MH), creatinine, glutamine, and acetate (Figure 3A–E). Urinary creatinine levels among these metabolites may be affected by renal function, but as described above, SCr and eGFR levels between cancer groups were confirmed to be normal, and this means that there were no metabolic differences according to renal function among groups.

4-HBA is a phenolic derivative of benzoate with anti-cancer properties [34]. Seidel et al. found that 4-HBA, a histone deacetylase-specific inhibitor, reduced the proliferation and viability of prostate cancer cells, and decreased androgen receptor levels, which play a role in the development and progression of prostate cancer [35]. Recently, in plasma metabolomic profiling by GC/MS, 4-HBA was found to discriminate between the control, prostatic intraepithelial neoplasia, and prostate cancer, and its levels increased with disease progression. In our study, 4-HBA had the highest concentration in PCa (Figure 3A), and based on previous studies, we explain that the levels of 4-HBA could be useful in determining the abnormal prostate metabolic profile [36].

*N*-MH is derived from creatinine-by-creatinine deaminase through an alternative creatinine-creatine pathway [37,38,39,40]. *N*-MH is further degraded to *N*-carbamoylsarcosine and sarcosine, and consequently to glycine. Several studies have explored the relation between the urinary levels of sarcosine and prostate cancer, which indicated increased sarcosine levels as a potential marker of prostate cancer [16,41,42,43]. Although other intermediates in the pathway for creatinine degradation to *N*-MH (e.g., *N*-carbamoylsarcosine, sarcosine) could not be detected in this study, we confirmed the highest median levels of *N*-methylhydantoin and glycine and lower levels of creatinine in PCa, suggesting that the metabolic pathway for creatinine degradation to *N*-MH could be a specific active pathway in prostate cancer cells (Figure 3B,C and Table S1).

Glutamine is converted to glutamate and pyroglutamate, which is produced by the cycling of glutamine involved in GSH metabolism [44,45]. Whenever there is an increased demand for GSH to defend against stress responses, this cyclic form of pyroglutamate is opened by 5-oxoprolinase to regenerate glutamate, which then produces GSH [46]. Ahmad et al. found an increased rate of synthesis of GSH from glutamine in papillary renal cell carcinoma tissues by proteomic and metabolomic analysis [47]. Similarly, we identified significantly lower levels of glutamine in RCC (Figure 3D and Table S1), suggesting that GSH synthesis from glutamine is promoted in renal cell carcinoma. 

As a metabolite relevant to major energy metabolism pathways, acetate, which is produced by glucose-derived pyruvate, supports acetyl-CoA metabolism and plays an important role in bioenergetics [48,49]. In tumor cells, acetate is mainly used as fuel for lipid synthesis by formed to acetyl-CoA [50]. Gao et al. reported that acetate, which is a crucial product of fatty acid *β*-oxidation, contributed to the discrimination of paired adjacent tissue from advanced RCC [51]. Furthermore, many previous studies have investigated an association between renal cell carcinoma and lipid metabolism, revealing that the lipid accumulation in tumor cell is the histological and morphological characteristics of RCC [52,53,54]. There is also evidence to suggest that lipogenesis is a feature of the malignant phase of RCC [55]. Based on the previous studies, we suggest that significantly high acetate levels in RCC could be supported to previous studies related to lipid metabolism in RCC (Figure 3E).

### 2.4. Evaluation of the Predictive Effectiveness of Significant Metabolites

To confirm the discriminative accuracy of individual metabolites between groups, we performed the univariate receiver operating characteristic (ROC) curve analysis using the prominent five metabolites (Figure 3). The criteria for assessing the accuracy of the signature based on the area under the curve (AUC), summarized into a single metric of ROC curve, were as follows: 0.9 to 1.0 = excellent; 0.8 to 0.9 = good to very good; 0.7 to 0.8 = fair; 0.6 to 0.7 = poor; 0.5 to 0.6 = fail [56]. Therefore, we found significant metabolites with AUC > 0.7 (*p* < 0.05) in each univariate ROC curve analyses (Table 2), and the multivariate ROC curves for intergroup were created by combining the significant metabolites in univariate ROC analysis and assessed for their predictability.

**Figure 4:**
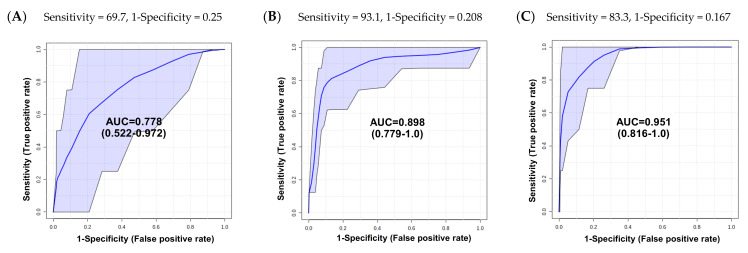
Combined multivariate ROC curves between cancer groups. (**A**) The AUC for creatinine and glutamine to discriminate BCa from RCC was 0.778 (95% CI, 0.522–0.972). (**B**) The AUC for 4-HBA, N-MH, and creatinine to discriminate PCa from BCa was 0.898 (95% CI, 0.779–1.0). (**C**) The AUC for 4-HBA, N-MH, glutamine, and acetate to discriminate PCa from RCC was 0.951 (95% CI, 0.816–1.0).

The multivariate ROC curve of BCa vs. RCC combined with creatinine and glutamine showed an AUC value of 0.778 with sensitivity = 69.7% and specificity = 75% (Figure 4A). The multivariate ROC curve with 4-HBA, N-MH, and creatinine showed with an AUC 0.898, sensitivity = 93.1%, specificity = 79.2% in classification between PCa and BCa (Figure 4B). The composition of 4-HBA, *N*-MH, glutamine, and acetate was able to accurately differentiate 95.1% of PCa from RCC, with high sensitivity (83.3%) and specificity (83.3%) (Figure 4C). The all-multivariate ROC curves between BCa, PCa and RCC showed high performance with empirical *p*-values less than 0.05 (Figure S2A–C), suggesting that these multivariate ROC findings can be used to determine which cancer is under BCa, PCa, and RCC.

## 3. Materials and Methods

### 3.1. Reagents

Deuterium oxide (D_2_O, 99% in D) was purchased from Cambridge Isotope Laboratories, Inc., (Andover, MA, USA). Potassium phosphate dibasic (K_2_HPO_4_, ACS grade reagent), sodium phosphate monobasic (anhydrous), hydrochloric acid (HCl, ACS grade reagent), sodium hydroxide solution (NaOH, HPLC grade), and 3-trimethylsilyl propionic-2,2,3,3-d4 acid sodium salt (TSP-d_4_) were obtained from Sigma-Aldrich (St. Louis, MO, USA).

### 3.2. Sample Collection

A total of 65 patients were recruited from Pusan National University Hospital (PNUH), including 29 with bladder cancer, 24 with prostate cancer, and 12 with renal cancer. The study was approved by the Institutional Review Board (IRB) of PNUH (IRB No 1802-004-063). Urine samples were collected during outpatient follow-up or prior to urological surgery. At the outpatient department, patients were asked to take mid-stream urine in an aseptic container. In the operating room, urine samples were acquired in an aseptic container via catheterization using 7 Fr. Nelaton catheter under general anesthesia. Before catheterization, preoperative skin preparation was conducted on the external genital organs using povidone-iodine solution. All the samples were over 50 mL in volume and immediately stored in a −80 ℃ freezer.

### 3.3. Preparation of Urine Sample for NMR Analysis

Urine samples for NMR analysis were prepared according to criterion procedures [57,58]. Samples were thawed at 25 ℃ (Room temperature), shaken before use, and 700 μL of each urine aliquot were centrifuged at 12,000× *g* for 15 min at 4 ℃ to precipitate protein and other macromolecules [45]. To minimize chemical shift variation described by Xiao et al. [59], 70 μL of K_2_HPO_4_ and NaH_2_PO4 buffer (pH 7.4, 1.5 M in D_2_O) containing 20 mM TSP-d_4_ was added to 630 μL of urine sample supernatant. Then, 700 μL of the mixture were transferred into a 5 mm NMR tube for NMR measurement.

### 3.4. NMR Spectra Acquisition and Spectral Processing

NMR measurements were conducted using an Agilent NMR spectrometer (Agilent, Santa Clara, CA, USA) equipped with a 600 MHz magnet at 298 K. All ^1^H NMR data collection and pre-processing were performed using VnmrJ Software (Agilent Technologies, Santa Clara, CA, USA). All NMR data of each sample were measured by a pulse sequence PRESAT in all cases to suppress the water signal during a relaxation time of 2 s and 90-degree pulse (pw) of 11.4 μs at 128 scans into 32 K data points. NMR data were obtained in the form of FID files, and the spectrum was acquired by Fourier transformation. The baseline and phase of the spectrum were manually corrected, and all spectra were calibrated with reference to the TSP-d_4_ signal at δ 0.00 ppm. The NMR spectrum segmented into buckets of 0.005 ppm with a range of 0.5 to 8.5 ppm was analyzed after excluding the water and urea peak region (4.2 to 6.1 ppm) and normalized to total area to minimize the urinary dilution effect among groups, then the peaks were aligned. NMR data were processed using Mnova (Mestrelab Research, Santiago de Compostela, Spain) because of the software suite’s useful functions such as peak binning and alignment, spectral normalization, and spectral superimposing. The peak signals of metabolites were identified and quantified using 600 MHz library from Chenomx NMR suite 8.4 professional (Chenomx Inc., Edmonton, AB, Canada), which could be obtained by comparing a known reference peak signal (TSP) with signals from a library of metabolites. In addition, to accurately confirm the identified compound signal, we also double-checked using data from public databases such as the human metabolome database (HMDB, www.hmdb.ca, accessed on 10 June 2021) [60] and Pubchem, and the literature [21]. The relative concentration of metabolites was analyzed to compare the significant differences among groups.

### 3.5. Statistical Analysis

To confirm the differences in the metabolic profile among urological cancers, multivariate pattern recognition analysis was performed for all processed NMR spectrum data using the SIMCA-P 12.0.1 software (Umetrics, Umeå, Sweden). The PLS/OPLS-DA analysis was performed with pareto scaling to examine the possible minor metabolic differences among groups, and the VIP analysis was used to identify the metabolites contributing for group separation in OPLS-DA model. The quality of the model was assessed using the cross-validation parameters R^2^Y(cum), Q^2^ (cum) which is reported that Q^2^ > 0.5 is admitted for good predictability in the model [61]. Furtherly, to assess the reliability of these classification models, analysis of variance testing of *p*(CV-ANOVA) was performed and *p*-value under 0.05 was determined to be significant. Before examining the significantly differential levels of metabolites among groups, the distribution of normalized concentration was estimated by the Kolmogorov-Smirnov test, and the metabolites with abnormal distribution were conducted by a non-parametric analysis of covariance (Ranked-ANCOVA) by adjusting the covariate (age) as a potential confounding factor in metabolites data set. The post-hoc tests of a ranked ANCOVA were executed using with a Bonferroni correction method by adjusted *p*-value (*p* < 0.05). Statistical analyses for the quantities of metabolites were performed using IBM SPSS Statistics 25 (SPSS, Inc., Chicago, IL, USA). ROC analysis between intergroups was performed using a web server for metabolomics data analysis (MetaboAnalyst 5.0, https://www.metaboanalyst.ca, accessed on 12 July 2021) [62]. Firstly, univariate ROC analysis was examined, and AUC and *p*-values of each ROC curves were used to evaluate the predictability. Then to improve the discriminatory accuracy, multivariate ROC curves were plotted with false positive rate and true positive rate using combination of significant metabolites with AUC > 0.7 (*p*-value < 0.05).

## 4. Conclusions

In summary: the present study revealed the different urinary metabolic profiles of bladder cancer (BCa), prostate cancer (PCa), and renal cell carcinoma (RCC) based on the ^1^H NMR spectroscopy with multivariate pattern recognition analysis and quantitative analysis. For seeking the prominent metabolites, several criteria were applied: (i) the metabolites with VIP value > 1.0 in the OPLS-DA model; (ii) ranked-ANCOVA with a Bonferroni-adjusted *p*-value < 0.05. Consequently, a total of five metabolites were identified as significant. These metabolites were used in univariate and multivariate ROC curve analysis to examine their sensitivity and specificity in discriminating among cancer groups. Multivariate ROC curves created by combining each significant metabolites for BCa vs. RCC (including of creatinine, and glutamine), PCa vs. BCa (including 4-hydroxybenzoate, *N*-methylhydantoin, and creatinine), and RCC vs. PCa (including 4-hydroxybenzoate, *N*-methylhydantoin, glutamine, and acetate) showed better discriminatory accuracy than univariate ROC model, suggesting that these multiple metabolites panel could be determinant of the urological cancers. Although this preliminary study demonstrated a comparison of holistic metabolic profiles between the urological cancers and supports the explorative role of metabolomics in the investigation of human urine, it is limited in that the sample size is small. Therefore, further experiments should be performed with more subjects with matched clinical characteristics (e.g., stage of cancer, age, etc.), including healthy controls. Through further study, we expect to provide a faster determination of urological malignancy types in patients with voiding dysfunction.

## Figures and Tables

**Table 1:** Summary of the clinical characteristics of the patients with BCa, PCa and RCC.

Characteristics	Variables	BCa (*n* = 29)	PCa (*n* = 24)	RCC (*n* = 12)
**Age (years)**		67.92 ± 10.42	68.21 ± 5.54	62.17 ± 11.76
**Sex (%)**	Male	29 (100)	24 (100)	12 (100)
**SCr** **(mg/dL)**		0.98 ± 0.28	0.79 ± 0.13	0.98 ± 0.19
**eGFR (mL/min/1.73 m^2^)**		81.81 ± 21.11	101.18 ±19.49	78.30 ±14.98
**pT stage (%)**	Ta	13 (44.83)	-	-
	Tis	2 (6.89)	-	-
	T1	11 (37.93)	3 (12.5)	7 (58.33)
	T2	3 (15.15)	6 (25.0)	-
	T3	-	14 (58.33)	4 (33.33)
	T4	-	1 (4.17)	1 (8.33)
**WHO/ISUP** **classification (%)**	Low	15 (48.27)	-	-
	High	14 (51.72)	-	-
**GS (%)**	6	-	5 (20.83)	-
	7	-	3 (12.50)	-
	8	-	8 (33.33)	-
	Above 8	-	8 (33.33)	-
**HSPC/CRPC (%)**		-	8 (33.3)/16 (66.7)	-
**Fuhrman grade (%)**	G2	-	-	8 (66.67)
	G3	-	-	2 (16.67)
	G4			2 (16.67)

Abbreviations: SCr, Serum creatinine; eGFR, estimated glomerular filtration rate; pT, Pathologic Stage; GS, Gleason Score; HSPC, Hormone Sensitivity Prostate Cancer; CRPC, Castration Resistant Prostate Cancer. Note: Data are presented as mean ± standard deviation. eGFR was calculated using by MDRD formula.

**Table 2:** Significance assessment of univariate receiver operating characteristic (ROC) curve analysis for metabolites potentially distinguishing between BCa, PCa, and RCC.

Metabolites	BCa vs. RCC			PCa vs. BCa			PCa vs. RCC		
	AUC	*p*-Value	95% CI	AUC	*p*-Value	95% CI	AUC	*p*-Value	95% CI
4-HBA	0.583	0.666	0.401–0.749	0.731	0.002	0.718–0.940	0.708	0.041	0.517–0.892
N-MH	0.686	0.044	0.500–0.872	0.840	8.875 × 10^−6^	0.717–0.946	0.917	1.667 × 10^−5^	0.824–1.0
Creatinine	0.714	0.007	0.486–0.914	0.740	0.002	0.587–0.856	0.549	0.338	0.22–0.714
Glutamine	0.815	2.054 × 10^−4^	0.655–0.947	0.672	0.024	0.522–0.81	0.924	1.364 × 10^−5^	0.82–0.989
Acetate	0.739	0.054	0.574–0.904	0.617	0.345	0.454–0.774	0.848	0.010	0.691–0.962

## Data Availability

Data are contained within the article or supplementary.

## Supplementary Materials

The following are available online at https://www.mdpi.com/article/10.3390/metabo11090591/s1, Figure S1: Orthogonal partial least-squares discriminant analysis (OPLS-DA) score plot between inter cancer groups, Figure S2: Empirical *p*-value plots of multivariate ROC curve analysis. Table S1: Identified and quantified urinary metabolites from ^1^H NMR spectra.
