# Supplementary material for: A Unique Urinary Metabolic Feature for the Determination of Bladder Cancer, Prostate Cancer, and Renal Cell Carcinoma
Source: Metabolites. 2021 Sep 2;11(9):591. doi: 10.3390/metabo11090591 (PMC8468099; doi:10.3390/metabo11090591)
Supplement: Supplementary file 1 [file metabolites-11-00591-s001.zip › [R2] Revised-Supplementary Figure_Urological cancer-Metabolomics.pptx]

## Slide 1
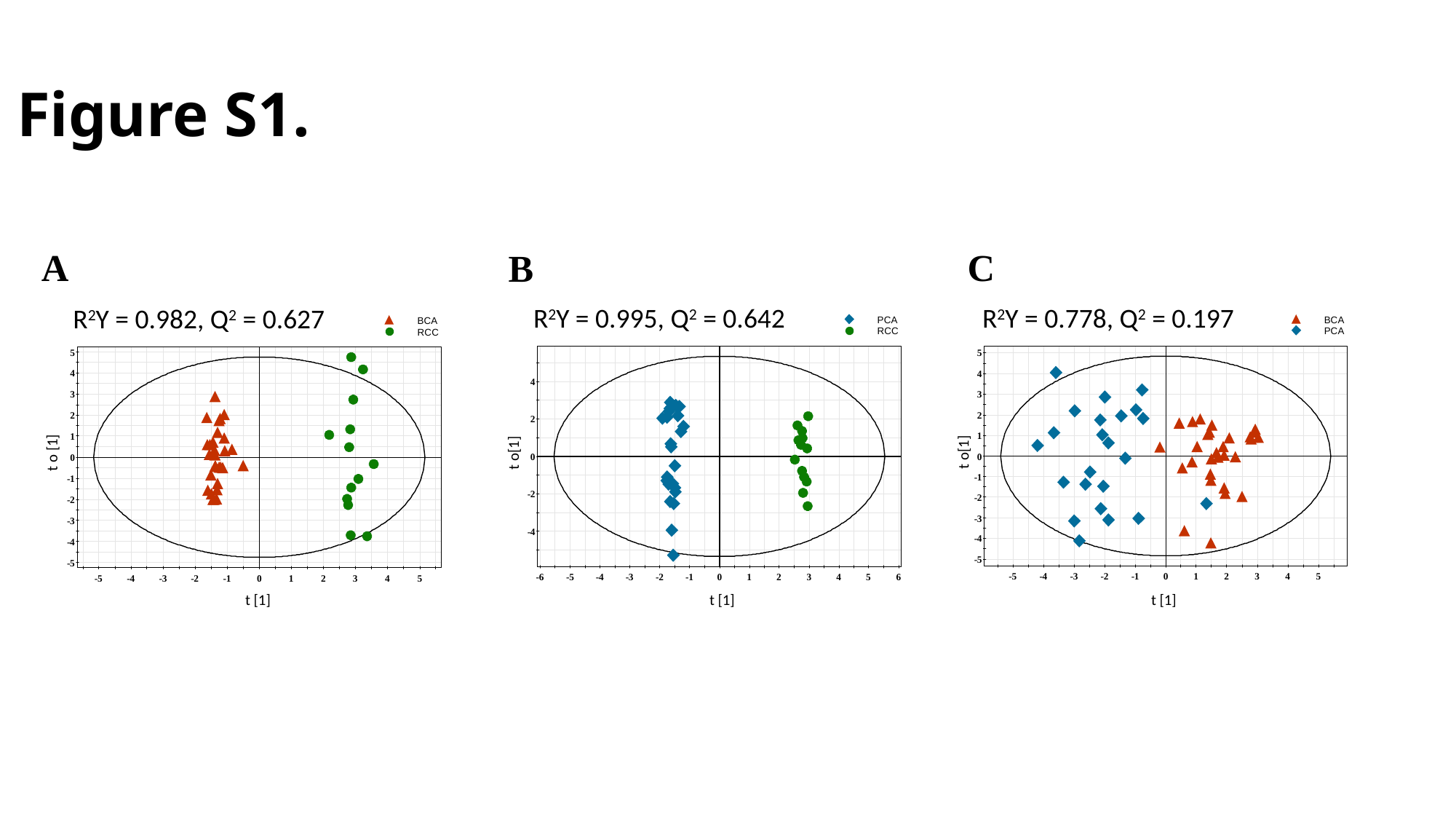

# Figure S1.
C
A
B
R2Y = 0.995, Q2 = 0.642
t o[1]
t [1]
R2Y = 0.778, Q2 = 0.197
t o[1]
t [1]
R2Y = 0.982, Q2 = 0.627
t o [1]
t [1]

## Slide 2
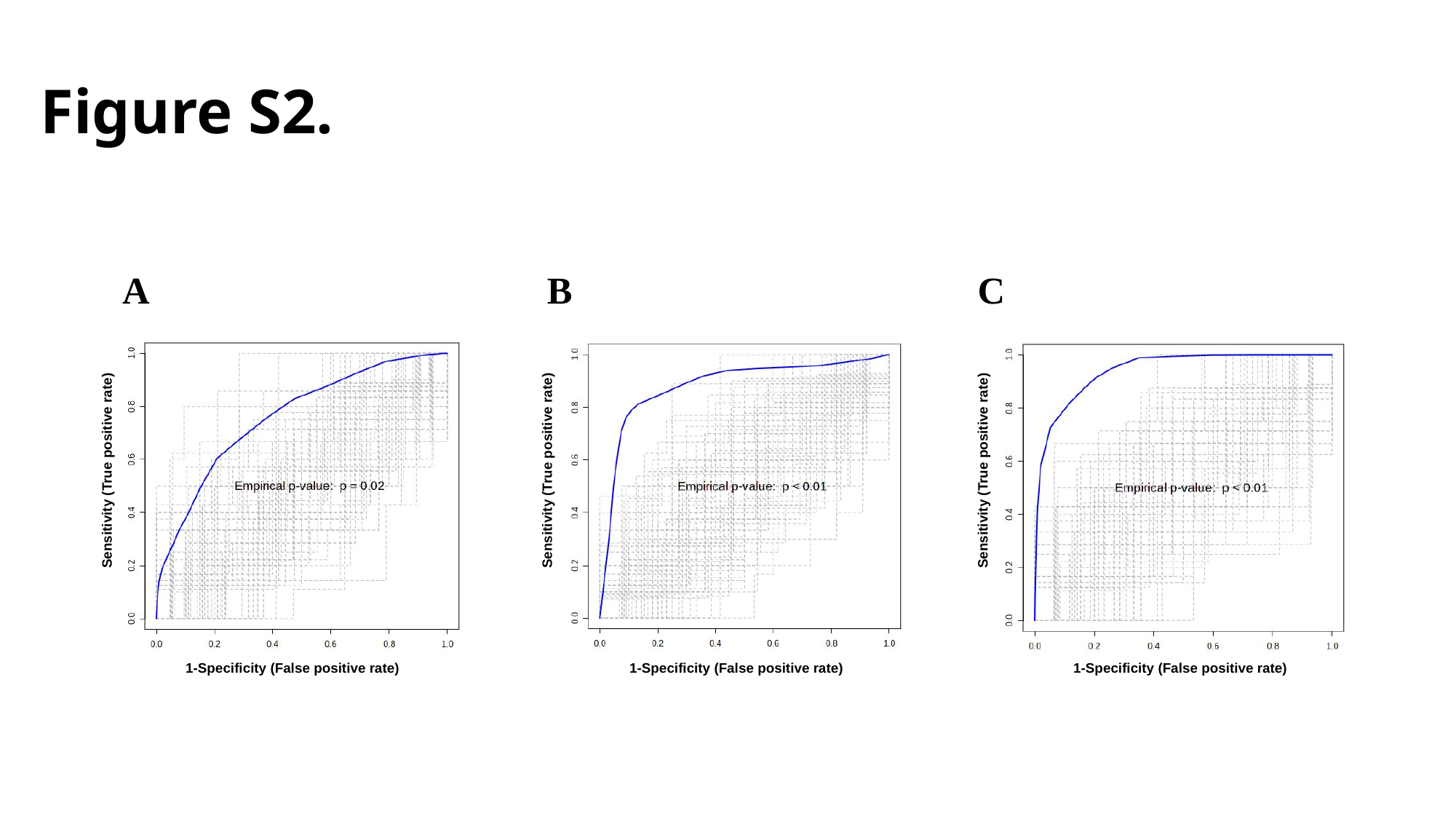

# Figure S2.
A
B
C
Sensitivity (True positive rate)
1-Specificity (False positive rate)
Sensitivity (True positive rate)
1-Specificity (False positive rate)
Sensitivity (True positive rate)
1-Specificity (False positive rate)
