# Supplementary material for: A Unique Urinary Metabolic Feature for the Determination of Bladder Cancer, Prostate Cancer, and Renal Cell Carcinoma
Source: Metabolites. 2021 Sep 2;11(9):591. doi: 10.3390/metabo11090591 (PMC8468099; doi:10.3390/metabo11090591)
Supplement: Supplementary file 1 [file metabolites-11-00591-s001.zip › [R2] Revised_Supplementary Table_Urological cancer-Metabolomics.pdf]

Table S1. Identified and quantified urinary metabolites from ^{1}H-NMR spectra. Values are median (mM) and IQR (Interquartile range) of normalized concentration. Statistical analyses of metabolites levels were conducted by using Kolmogorov-Smirnov test for normality and non-parametric analysis of covariance followed by Bonferroni correction method for significant differences among groups.

Metabolites	BCa			PCa			RCC			Significance (adjusted *p*-value)	Multiple Comparison Analysis
	Median	IQR	Normality (*p*-value)	Median	IQR	Normality (*p*-value)	Median	IQR	Normality (*p*-value)		
2-Hydroxyisobutyrate	0.041	0.024	2.00E-01	0.038	0.013	1.14E-02	0.043	0.033	2.00E-01	1.006	
3-Hydroxyisobutyrate	0.060	0.041	2.45E-02	0.051	0.028	5.57E-02	0.048	0.031	6.82E-02	0.181	
3-Hydroxyisovalerate	0.032	0.017	2.57E-07	0.029	0.012	2.90E-03	0.035	0.024	2.00E-01	1.412	
4-Hydroxybenzoate	0.055	0.063	7.98E-03	0.099	0.120	2.39E-03	0.059	0.050	1.14E-01	0.024*	B=R; B<P; R<P
4-Hydroxyphenylacetate	0.090	0.072	1.94E-09	0.094	0.128	7.16E-03	0.103	0.049	2.00E-01	0.487	
Acetate	0.067	0.035	6.20E-04	0.051	0.038	1.85E-04	0.102	0.023	1.14E-08	0.007**	B=P; B<R; P<R
Alanine	0.228	0.131	2.00E-01	0.24	0.135	8.04E-02	0.190	0.132	3.69E-02	1.941	
Arabinitol	0.447	0.272	1.15E-06	0.499	0.376	3.78E-05	0.456	0.156	2.00E-01	0.611	
Betaine	0.110	0.121	1.04E-03	0.097	0.081	1.25E-01	0.139	0.156	1.79E-02	0.261	
Carnitine	0.084	0.151	5.24E-02	0.042	0.071	4.86E-10	0.100	0.086	1.57E-02	1.192	
Choline	0.044	0.022	5.40E-12	0.041	0.026	7.40E-08	0.041	0.024	2.16E-09	2.805	
Citrate	2.732	2.528	2.00E-01	2.198	1.734	2.00E-01	2.406	1.928	1.32E-01	2.245	
Creatine	0.134	0.084	4.94E-10	0.173	0.171	1.19E-06	0.254	0.557	3.16E-04	1.908	
Creatinine	8.580	2.917	2.00E-01	6.601	2.302	3.85E-02	6.412	3.991	2.00E-01	0.002**	B>P; B>R; P=R
Dimethylamine	0.507	0.126	1.05E-01	0.448	0.190	2.00E-01	0.563	0.448	1.19E-01	0.295	
Formate	0.276	0.219	2.72E-02	0.252	0.182	3.05E-02	0.360	0.357	2.00E-01	0.912	
Gluconate	0.393	0.129	6.32E-12	0.400	0.132	1.57E-03	0.508	0.319	2.00E-01	0.724	
Glucose	0.343	0.219	3.92E-14	0.392	0.262	2.61E-11	0.456	0.297	2.87E-02	1.265	
Glutamine	0.189	0.100	4.30E-02	0.234	0.089	2.00E-01	0.137	0.073	2.00E-01	< 0.001***	B=P; R<B; R<P
Glycine	0.580	0.303	3.43E-07	0.808	0.441	2.00E-01	0.645	0.364	2.08E-02	0.280	
Glycolate	0.307	0.191	1.08E-01	0.296	0.124	7.61E-05	0.406	0.224	2.00E-01	2.407	
Hippurate	0.757	0.868	1.93E-02	0.688	1.235	3.42E-02	0.752	1.284	1.09E-01	2.951	
Histidine	0.458	0.230	2.00E-01	0.522	0.473	2.02E-02	0.357	0.174	9.90E-03	0.196	
Lactate	0.105	0.076	6.38E-11	0.104	0.071	2.00E-01	0.112	0.055	8.02E-02	0.577	
Lysine	0.104	0.089	8.53E-03	0.089	0.101	6.04E-03	0.086	0.060	2.00E-01	1.338	
Mannitol	1.065	0.751	1.59E-10	0.698	0.763	2.63E-04	0.430	0.646	9.20E-05	0.252	
Methionine	0.016	0.005	2.00E-01	0.014	0.004	1.33E-01	0.013	0.008	2.00E-01	0.795	
N, N-Dimethylglycine	0.019	0.024	4.35E-02	0.016	0.011	5.58E-05	0.018	0.030	3.64E-02	2.575	
N-Methylhydantoin	0.039	0.020	2.00E-01	0.075	0.046	5.50E-04	0.028	0.015	7.29E-02	< 0.001***	P>B; P>R; B=R
N-Phenylacetylglycine	0.175	0.083	5.96E-02	0.236	0.139	3.60E-03	0.194	0.107	2.00E-01	0.063	

Table S1. *Cont.*

Metabolites	BCa			PCa			RCC			Significance (adjusted *p*-value)	Multiple Comparison Analysis
	Median	IQR	Normality (*p*-value)	Median	IQR	Normality (*p*-value)	Median	IQR	Normality (*p*-value)		
O-Acetylcarnitine	0.025	0.033	1.60E-03	0.014	0.026	3.16E-07	0.024	0.021	2.00E-01	0.623	
Pseudouridine	0.125	0.040	2.00E-01	0.105	0.032	2.00E-01	0.121	0.064	2.00E-01	0.086	
Pyroglutamate	0.195	0.069	2.00E-01	0.151	0.079	1.25E-01	0.179	0.106	2.00E-01	0.087	
Succinate	0.027	0.032	8.91E-04	0.024	0.027	9.29E-03	0.028	0.024	1.54E-01	2.091	
Taurine	1.182	0.820	1.23E-01	1.856	1.369	2.00E-01	1.463	1.145	2.00E-01	0.358	
Trimethylamine *N*-oxide	0.799	0.777	1.13E-02	0.617	0.736	2.00E-05	2.275	1.965	9.76E-03	0.140	
Tyrosine	0.101	0.049	1.07E-05	0.085	0.022	2.00E-01	0.089	0.080	7.49E-02	0.940	
Valine	0.031	0.008	1.22E-03	0.029	0.008	2.00E-01	0.029	0.012	6.00E-02	0.568	
cis-Aconitate	0.187	0.099	2.00E-01	0.156	0.043	2.00E-01	0.181	0.048	2.00E-01	0.902	
myo-Inositol	0.170	0.163	1.03E-06	0.174	0.165	3.55E-02	0.260	0.292	2.00E-01	1.787	

* Significantly different with adjusted p-value < 0.05; ** Significantly different with adjusted p-value < 0.01; *** Significantly different with adjusted p-value < 0.001
